# Safranal Inhibits Angiogenesis *via* Targeting HIF-1α/VEGF Machinery: *In Vitro* and *Ex Vivo* Insights

**DOI:** 10.3389/fonc.2021.789172

**Published:** 2022-02-02

**Authors:** Ali Abdalla, Chandraprabha Murali, Amr Amin

**Affiliations:** ^1^ Weinberg Institute for Cognitive Science, University of Michigan, Ann Arbor, MI, United States; ^2^ Biology Department, College of Science, United Arab Emirates University, Al-Ain, United Arab Emirates; ^3^ The College, The University of Chicago, Chicago, IL, United States

**Keywords:** safranal, VEGF, HIF-1α, angiogenesis, cancer

## Abstract

Nature has a nearly infinite inventory of unexplored phytochemicals and biomolecules that have the potential to treat a variety of diseases. Safranal exhibits anti-cancer property and the present study explores its antiangiogenic property. Hepatocellular carcinoma (HCC) ranks as the sixth deadliest among all cancer types. Targeting the non-tumor vasculature supporting system is very promising as it has less plasticity, unlike malignant cells that are often associated with issues like drug resistance, poor prognosis, and relapse. In this study, we successfully inhibited the proliferation of primary human umbilical vein endothelial cells (HUVEC) with an IC50 of 300μM and blocked VEGF secretion in HepG2 cells. Furthermore, safranal inhibited VEGF-induced angiogenesis *in vitro* and *ex vivo via* scratch wound assay, tube formation assay, transmembrane assay, and aortic ring assay. In addition, safranal downregulated the *in vitro* expression of HIF-1α, VEGF, VEGFR2, p-AKT, p-ERK1/2, MMP9, p-FAK, and p-STAT3. The present study is the first to reveal the antiangiogenic potential of safranal and propose its possible underlying mechanism in HCC.

**Graphical Abstract:**
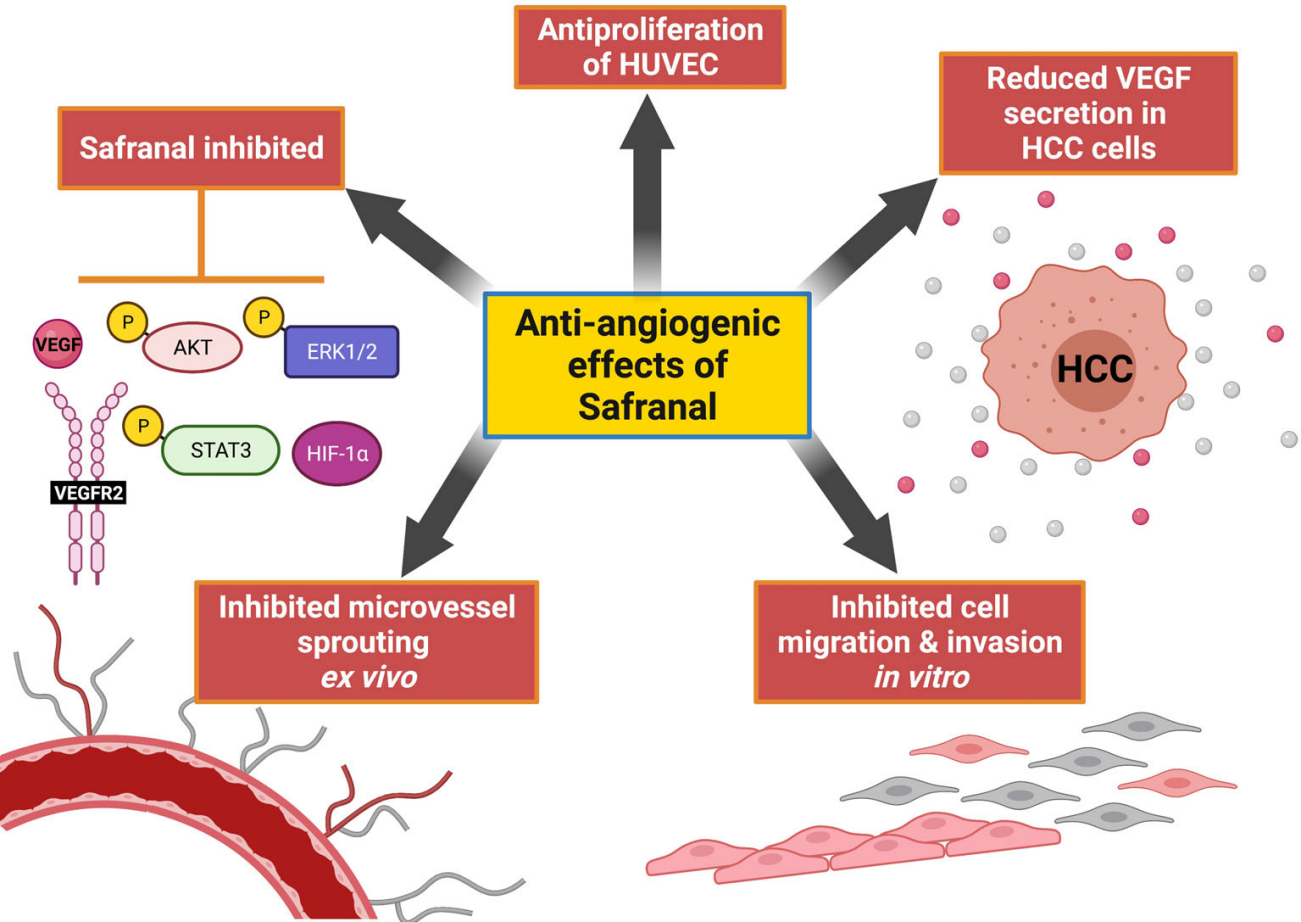
Safranal exhibited antiangiogenic properties in this study. Safranal shows pleiotropic effect by repressing HIF-1α which is a strong activator of VEGF. This could be due to the inhibition of ERK and AKT phosphorylation which tightly regulates HIF-1α synthesis. Finally preventing the activation of VEGF/VEGFR2 axis. Hence, aalteration of the key vascular endothelial signaling molecules are in favor of inhibiting the *in vitro* and *ex vivo* angiogenic assays. This figure was created with BioRender.com.

## Introduction

Globally, liver cancer is one of the most fatal cancers (1). Hepatocellular carcinoma (HCC), the most common type of primary liver cancer, ranks sixth deadliest among all cancer types (2). The high frequency of HCC can be traced back to a host of risk factors that often lead to the development of HCC. Viral hepatitis infections, specifically with hepatitis B virus (HBV) and hepatitis C virus (HCV), alcoholism, smoking, nonalcoholic fatty liver disease, chronic liver disease, and cirrhosis represent some of the major risk factors (3). Many forms of cancer, including HCC, cause the deregulation of multiple signaling pathways that manage cell proliferation, metastasis, and angiogenesis (4). Neovascularization is a crucial event in tumor progression from the sprouting phase to more aggressive metastasis (5). Once fully developed, a solid tumor can remain latent if deprived of its blood supply (6). A thorough study of new blood vessels establishing in the tumor microenvironment is a promising prognostic marker both for grading the tumor and determining proper therapy for cancer patients (7). Inhibition of angiogenesis as an anti-cancer therapy was first hypothesized by Folkman in 1971 (8). Under normal conditions, angiogenesis is a tightly regulated physiological process. It is essential in wound healing, embryogenesis, and other vital processes in growth and development (9). Epidermal growth factor (EGF), insulin-like growth factor (IGF), and vascular endothelial growth factor (VEGF) are the most frequently studied signaling molecules in angiogenesis. Overexpression of these growth factors, particularly VEGF and its receptors, has been widely reported in HCC patients (10).

Typically, surgery would be the first treatment option for HCC. However, as the majority of patients are not eligible candidates for surgery at the time of their diagnosis, HCC therapeutics have significantly developed over recent years. Along with a growing list of novel curative agents and molecular targets, liver-directed, systemic, and immuno-therapy treatments have been the center of attention in HCC treatment (11). Many of these novel agents are natural-product based compounds that possess potent anticancer properties that can overcome chemoresistance and offer effective therapeutic and preventive alternatives with higher safety margins and minimal adverse effects (12). The use of these biomolecules in conjunction with other therapies has the potential to bring cancer to heel. With the ability to block fibrogenesis, suppress tumorigenesis, and inhibit oxidative stress in the liver, medicinal biomolecules have gained a great deal of momentum as an effective and affordable modality to treat chronic liver diseases across the globe (13).

Nutraceuticals are food components responsible for physiological and metabolic functions and they have been known to protect against a variety of chronic diseases. Their active biomolecules are known to promote health and can be used to prevent or treat a variety of ailments (14). Interestingly, a vast spectrum of therapeutic properties that include antidiabetic, anti-inflammatory, antioxidant, cardioprotective, antidepressant, antitussive, antitumor, and anticonvulsants have been attributed to special types of nutraceuticals derived from saffron - the stigma of *Crocus sativus* L (15). Crocus sativus L. is a perennial valuable medicinal food herb (Iridaceae family) that has been used in folk medicine and has a great exporting importance in Iran and India (16). Saffron and its fundamental components have been shown to have no cytotoxic effects on normal cells while still proving to be lethal to cancer (17–21). Studies have proved that saffron and its constituents suitably act against cancer development and show selective toxicity against tumors (22, 23). The precise mechanism of saffron’s anti-cancer properties remains elusive, but a few hypotheses have been drawn in these studies.

Due to the hypervascular nature of HCC, angiogenesis plays a key role in its progression. In this study we investigate the anti-cancer potential of safranal with a special interest in its anti-angiogenic capacity.

## Materials and Methods

### Cell Culture

Liver cancer cells, HepG2 (ATCC HB-8065), were cultured in RPMI 1640 medium (Hyclone, USA) and 1% of 100 U/ml penicillin and 100 μg/ml streptomycin (Sigma, USA) supplemented with 10% FBS (Sigma, USA). HUVEC (CLS, CRL-1730) cells were cultured in endothelial cell growth medium (ECGM) containing 20% FBS, at 37 °C in a humidified 5% CO_2_ atmosphere. Cells were sub-cultured for 2-4 days using trypsin 0.25%-EDTA (Hyclone, USA).

### Cell Viability

Cell proliferation assay on HepG2 and HUVEC cells were done as described in Al-Hrout et al. (17). Briefly, cells were seeded in triplicate at a density of 5000 cells/well in 96-well plates and grown in 100μl of complete growth medium and allowed to grow for 24 hours. Cells were then treated with various concentrations of safranal (Sigma-Aldrich, USA) (300, 500, 700 μM) and incubated for 24 hrs with or without recombinant 30 ng/ml human VEGF (rhVEGF) (Abcam). After the incubation period, cell viability was assessed using the CellTiter-Glo luminescent cell viability assay kit according to manufacturer instructions (Promega, WI).

### VEGF ELISA Assay

The presence of VEGF in cell culture media with and without safranal treatment was assessed by the ELISA kit (SIGMA) according to the manufacturer’s instructions. Each sample was analysed in triplicate.

### HUVEC Cell Wound Scratch Assay

HUVEC cells were seeded in a six-well plate in complete medium (ECGM, Sigma) and allowed to grow into a 70-80% confluence monolayer. The monolayer was then scratched with a new 10μl pipette tip across the centre of the well. After scratching, the detached cells were removed by gently washing the well with culture medium. Media containing 0.5% FBS was added with 30 ng/ml rhVEGF along with, or without, different concentrations of safranal. The area of the wound was photographed randomly at 0 h, 8 h, and 12 h and the wound area was measured, considering rhVEGF-induced untreated control wells as 100%.

### Western Blotting

HepG2 cells were seeded at a density of 1X10^6^ cells/100 mm plate and allowed to attach. Cells were then treated with increasing concentrations of safranal (300, 500, 700µM) for 24 hours. Whole cell lysates were separated using 10-15% SDS polyacrylamide gel electrophoresis. Proteins were transferred onto PVDF membranes prior to incubation with various primary antibodies; MMP9, AKT, p-AKT, p-FAK, p-ERK1/2, ERK1/2, p-PLCγ, p-STAT3, STAT3 (Cellsignalling technologies), and GAPDH (Abcam) were used as loading controls. As secondary

Antibodies, anti-mouse IgG (FC) peroxidase antibody (Cellsignalling technologies, 1:2000) and anti-rabbit IgG peroxidase antibody (Cellsignalling technologies, 1:2000) were used. Protein bands were detected using WesternSure Chemiluminescent Substrate (LI-COR) and C-DiGit blot scanner (LI-COR).

### Immunocytochemistry and Fluorescent Staining

HepG2 cells were seeded at a density of 3 X 10^4^ cells/well in an 8-chambered glass plate and allowed to attach before being treated with the most effective concentrations of safranal for 24 hours. Cells were then fixed with 4% paraformaldehyde followed by incubation with primary antibody for VEGFR2 (Cellsignalling technologies) and with secondary antibodies tagged with FITC (Alexa Fluor, Molecular Probes). Finally, the nuclei were stained using 4, 6-diamidino-2-phenylindole (DAPI; 0.5 μg/mL in PBS; for 5 min at room temperature). Cells were imaged using an Inverted Phase Contrast Microscope, model IX53, with a fluorescent attachment complete with the Olympus microscope high resolution digital camera, and model PD73.

### Transwell Migration Assay

Transwell invasion assay was done as previously described (24). Briefly, to the bottom chambers of the transwell plate (Corning), serum-free medium containing rhVEGF (30 ng/ml) was added. HUVECs were trypsinised and suspended with serum-free medium and 1 × 10^5^ cells per well were seeded into the top chambers of the transwell plate coated with, or without, extracellular matrix (ECM) in the presence, or absence, of safranal at stated concentrations. The transwell plate was incubated in a 5% CO_2_ incubator at 37°C for about 8-10 hrs. After the incubation, non-migrated cells on the surface of the membrane were wiped with a cotton swab and the invasive cells located on the bottom membrane were fixed with cold 4% paraformaldehyde for 30 min and stained with crystal violet solution or the nuclear stain DAPI. Images were taken using the Inverted Phase Contrast Microscope, model IX53, with a fluorescent attachment complete with the Olympus microscope high resolution digital camera model PD73.

### Rat Aorta Ring Assay

The present study was approved by the institutional (UAE University) Animal Ethics Committee (approval Reference number: A 8-15). This assay was carried out on rat aortic explants as previously described in Al-Salahi et al. and Al-Dabbagh et al. (25, 26). Thoracic aortas were removed from 3% sodium pentobarbital -euthanized male rats, rinsed with serum free medium, and cleaned from fibro adipose tissues. In total, 10 rats were used in this assay and the aortas were cross sectioned into small rings (each ring is about 1 mm thickness). The rings were seeded individually in 48-wells plate in 300μL serum free M199 media containing 3 mg/ml fibrinogen and 5 mg/ml aprotinin. Ten microliters of thrombin (50 NIH U/ml in 1% bovine serum albumin in 0.15 M NaCl) was added into each well and incubated at 37°C for 90 min to solidify. A second layer (M 199 medium supplemented with 20% HIFBS, 0.1% έ-aminocaproic acid, 1% L-Glutamine, 2.5μg/ml amphotericin B, and 60μg/ml gentamicin) was added into each well (300μL/well). All the extracts were added at final concentrations of 100μg/ml. On day two, the medium was replaced with a fresh one containing safranal at 500 µm. Aortic rings were photographed on day 2, 4, 6, and 8 using an Inverted Phase Contrast Microscope, model IX53, with the Olympus microscope high resolution digital camera model PD73. Subsequently, the length of the blood vessels outgrowth from the primary tissue explants was measured using Leica Quin software.

The inhibition of blood vessels formation was calculated using the formula:


% blood vesselsinhibition = [1− (A0/A)] × 100

Where;

A0 = distance of blood vessels growth in treated rings in μm and A = distance of blood vessel growth in the control in μm.

### Tube Formation Assay

96-well plate was coated with 50μl of growth factor reduced Corning Matrigel matrix (Corning Lifesciences, USA) according to manufacturer’s protocol. The plate was then incubated at 37°C for 45 min to solidify the Matrigel. HUVEC cells were seeded (2× 10^4^) on the top of the Matrigel in 100μl serum free culture medium with, or without Safranal under the stimulation of rhVEGF (30 ng/ml). 6-8 hrs later, tubular structures of endothelial cells and extend of network formation mimicking angiogenesis were examined using the Inverted Phase Contrast Microscope, model IX53, with the Olympus microscope high resolution digital camera model PD73. The number of the tubes was quantified from three random fields.

### Quantitative Real-Time PCR (qPCR)

For qPCR, cDNA corresponding to 50 ng of total RNA was used per transcript to be quantified. Quantitative PCR reactions were performed on an Applied Biosystems instrument system using the GoTaq^®^ qPCR Kit (PROMEGA, USA) with gene-specific primers according to the manufacturer’s instructions. Data was normalized using housekeeping gene averages for the same time point and condition (ΔCt). Values are shown as fold change relative to the untreated control (RQ). The primers for the qPCR reactions are listed in **Supplementary Table 1**.

### Statistical Analysis

All experiments were conducted in replicates. The quantitative data were shown as Mean ± SD and the statistical differences between two groups was examined by a two-tailed Student’s t test. p< 0.05 indicated the significant difference.

## Results

### Safranal Inhibits VEGF-Induced Angiogenesis

**Figure 1:**
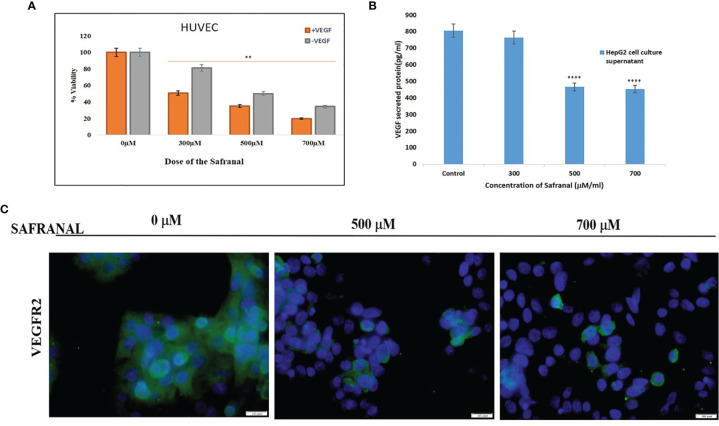
Safranal supressed growth by antagonising the tumor angiogenic proteins: **(A)** Safranal inhibited VEGF-induced HUVECs proliferation. Assessed viability of HUVEC cells that were serum starved overnight and then incubated with or without VEGF (30 ng/ml) and various concentrations of safranal for 24 hrs. **(B)** Safranal suppressed VEGF secretion in HepG2 cells. HepG2 cells were treated with various concentrations of safranal for 24 hrs, the supernatant of cell culture medium was collected, and the content of VEGF was examined using ELISA. **(C)** Immunofluorescence staining of HepG2 treated with various concentrations of safranal for 24 hrs, with rhVEGF (30 ng/ml) and then staining with VEGF (green), nuclei labelled by DAPI (blue). Scale bars: 100μm. Statistical analysis was carried out in all experiments by student’s t-test using GraphPad Prism software and p < 0.05 was considered as statistically significant. **p < 0.01 and ****p < 0.0001.

Based on our earlier study on the HCC cells (17), and in order to further assess its antiangiogenic potential, we examined the inhibitory effect of safranal on cell viability in HUVEC cells. Interestingly, safranal inhibited cell growth at a dose of 300 μM attaining IC50 on rhVEGF induced HUVECs compared to the non-induced cells (**Figure 1A**). The pro-angiogenic tumor derived factor, vascular endothelial growth factor (VEGF), was assessed after safranal treatment. HepG2 cells were treated with various concentrations of safranal for 24 hrs and the supernatant of the cell culture medium was collected and then secreted VEGF was examined using ELISA. As shown in **Figure 1B**, safranal reduced the levels of VEGF secreted by HepG2 cells in a dose dependent manner. After 24 hrs, there was nearly a 70% reduction in VEGF secretion from treated cells as compared to untreated control cells. We then assessed the effect of safranal on the highly expressed VEGF receptor in HCC, VEGFR2. Immunofluorescence analysis showed that the expression of VEGFR2 was reduced in HepG2 cells upon safranal treatment at higher doses of 500µM and 700µM (**Figure 1C**). There was not much difference in VEGFR2 expression at 300µM compared to control (data not shown). These results encouraged us to further investigate the effect of safranal on the VEGF/VEGFR2 signalling pathway.

### Safranal Inhibits the Migration and Tube Formation of Endothelial Cells

**Figure 2:**
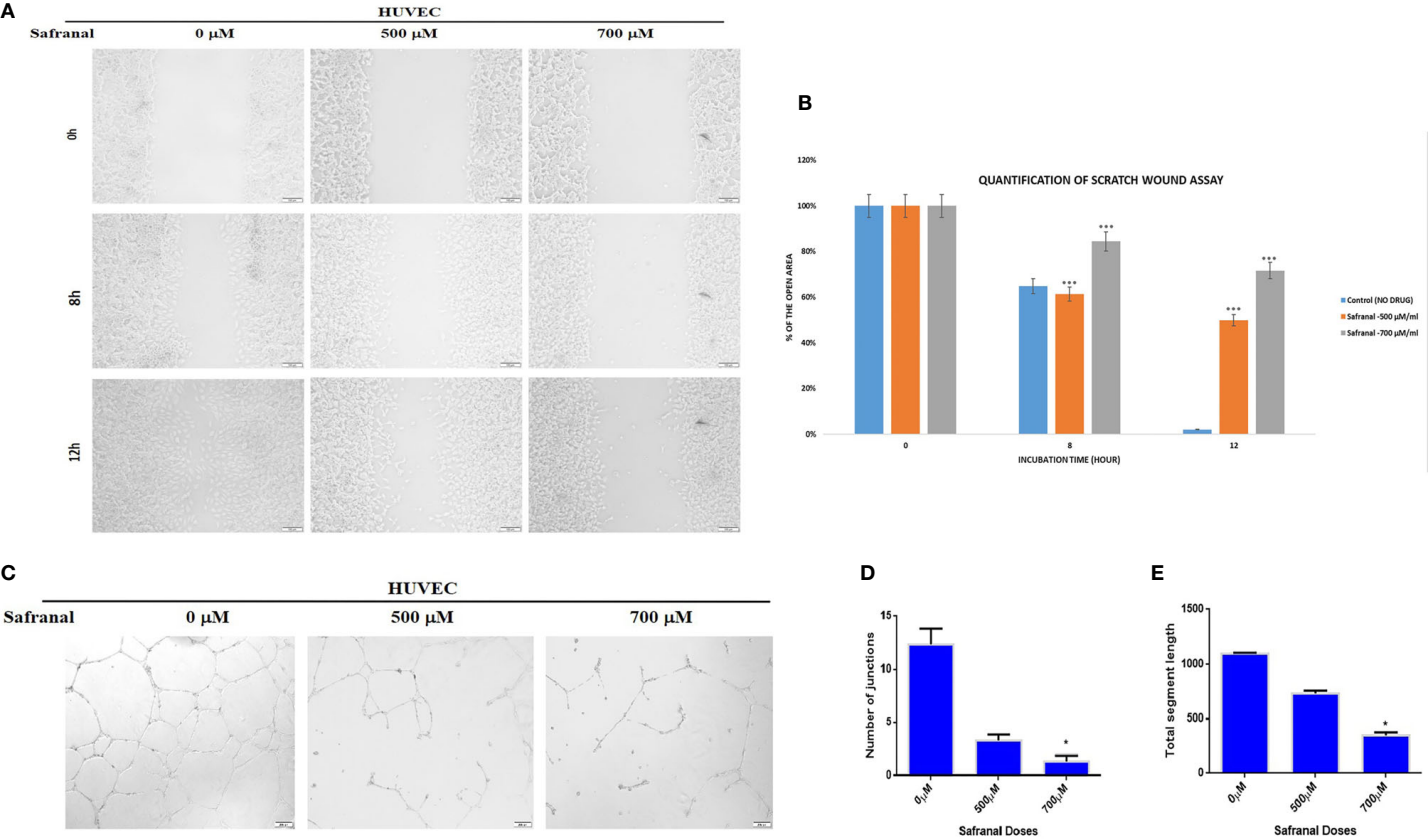
Safranal inhibited HUVECs migration and tube formation. **(A)** HUVECs were grown into full confluence in six-well plate, then cells were wounded with pipette. Further treated with 30 ng/ml rhVEGF as well as various concentrations of safranal. **(B)** The area of the wound was measured at 0 hr, 8 hrs and12 hrs. **(C)** HUVECs incubated with different concentrations of safranal were seeded into 96-well plate pre-coated with Matrigel. After 6 to 8 hrs, tubular structures were photographed, and the number of the tubes was quantified **(D, E)**. Statistical analysis was carried out in all experiments by student’s t-test using GraphPad Prism software and p < 0.05 was considered as statistically significant. *p < 0.05 and ***p < 0.001.

In order to study the effect of safranal on cellular migration and angiogenesis *in vitro*, wound healing assay and matrigel tube formation assay were performed in HUVECs. As shown in **Figure 2A**, a scratch wound was made in HUVEC cells which was followed by safranal treatment with rhVEGF induction. The wound area at 0 h was considered as 100% during the quantitative analysis (**Figure 2B**) and safranal inhibited the rhVEGF induced HUVEC migration thereby preventing the wound from healing in a dose dependent manner. After 12 hrs, the untreated, rhVEGF induced, HUVEC cells migrated and closed up the wound, yielding a nearly 0% wound area, while safranal proved effective, especially at 700 μM, where the wound area remained almost more than 60% open. In the matrigel tube formation assay, untreated endothelial cells formed tubes when induced with rhVEGF, while those in the presence of safranal failed to sprout despite rhVEGF induction. As shown in **Figure 2C**, the tube-like structures affect decreased at the doses of 500 μM and 700 μM of safranal and the significance was determined by counting the number of junctions and segment lengths of the modelled neovascularization (**Figures 2D, E** **)**. There was no significant difference in 300 μM treated cells compared to control (data not shown)

### Safranal Suppresses VEGF Induced Cell Invasion

**Figure 3:**
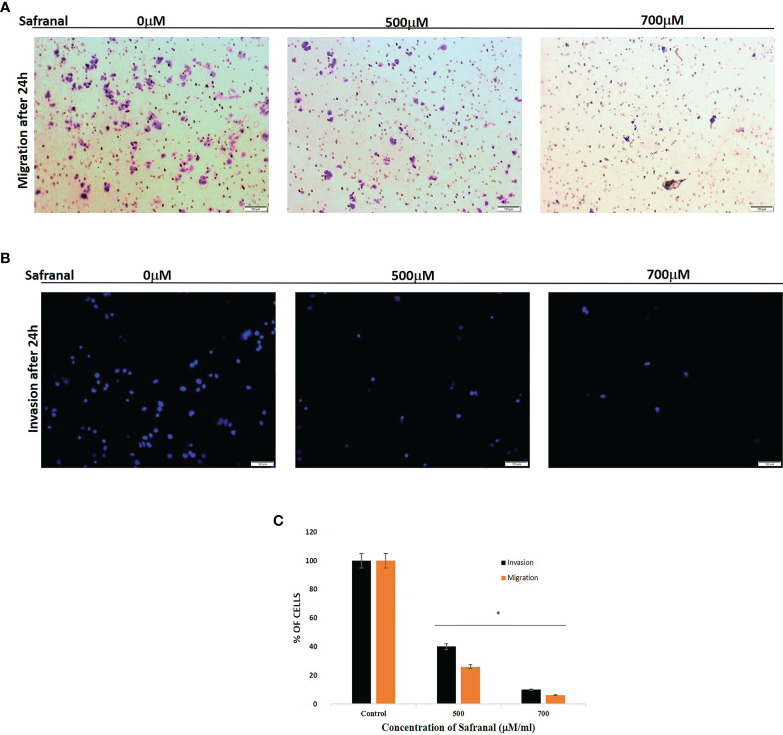
Safranal suppressed HUVECs invasion. Cells were seeded in the upper chamber of Transwell coated with matrigel and incubated with various concentrations of Safranal. The bottom chamber was added with culture medium with 30ng/ml rhVEGF. 24 hrs later, the nucleus of the migrated or invaded cells were stained with crystal violet **(A)** or DAPI **(B)**. The cells were quantified **(C)** through manual counting and presented as the mean ± standard deviation of three independent experiments performed in triplicate. Statistical analysis was carried out by student’s t-test using GraphPad Prism software and p < 0.05 was considered as statistically significant. *p < 0.05.

As safranal showed a significant effect in blocking the wound healing and tube formation in HUVEC, we proceeded to examine the antiangiogenic effect of safranal *via* transwell invasion assay on non-endothelial, HCC cells, HepG2. HepG2 cells were seeded in the upper chamber of transwell coated with (invasion), or without (migration), matrigel and incubated with various concentrations of safranal. The bottom chamber was added with culture medium containing 30ng/ml of rhVEGF. After 24 hrs, the nuclei of the invaded (**Figure 3B**) and migrated (**Figure 3A**) cells through the membrane were stained with DAPI/crystal violet respectively. Images of these cells were captured using a fluorescence microscopy in five random fields. The number of migrating and invading cells evidently decreased at a dose of 500 μM and 700 μM of safranal. The relative migration and invasion were quantified and analysed compared to untreated control cells (**Figure 3C**). There was no significant difference in 300 μM treated cells compared to control (data not shown)

### Safranal Blocks Angiogenesis in *Ex Vivo* Setting

**Figure 4:**
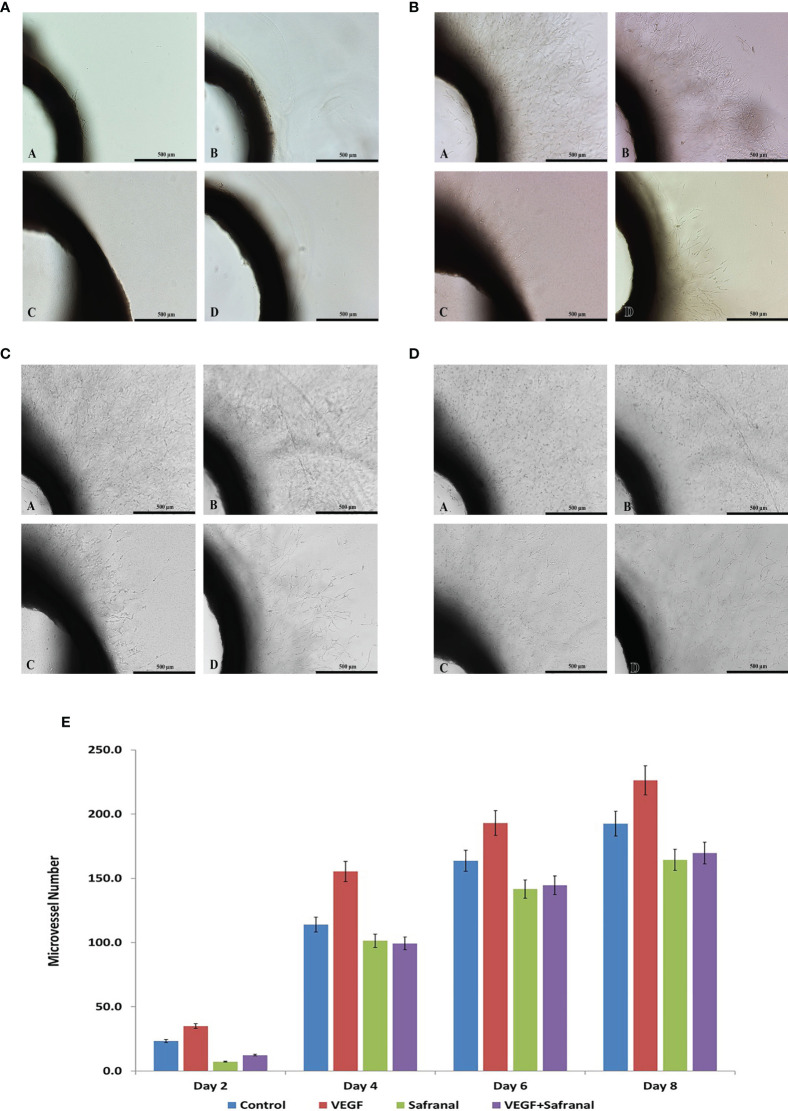
Safranal inhibits angiogenesis *ex vivo*. **(A)** Effects of safranal on microvessels sprouting in aortic ring assay two days post treatment. Representative micrographs of sprouting microvessels from aortic ring grown in the absence **(a)** or presence **(c)** of safranal with VEGF added alone **(B)** or with tested drug **(d)**. **(B)** Effects of safranal on microvessels sprouting in aortic ring assay four days post treatment. Representative micrographs of sprouting microvessels from aortic ring grown in the absence **(a)** or presence **(c)** of safranal with VEGF added alone **(b)** or with tested drug **(d)**. **(C)** Effects of safranal on microvessels sprouting in aortic ring assay six days post treatment. Representative micrographs of sprouting microvessels from aortic ring grown in the absence **(a)** or presence **(c)** of safranal with VEGF added alone **(b)** or with tested drug **(d)**. **(D)** Effects of safranal on microvessels sprouting in aortic ring assay eight days post treatment. Representative micrographs of sprouting microvessels from aortic ring grown in the absence **(a)** or presence **(c)** of safranal with VEGF added alone **(b)** or with tested drug **(d)**.

The present study shows the inhibitory effect of safranal on angiogenesis in rat aortic explants. The antiangiogenic effect of safranal was measured in the presence of VEGF in a time dependent manner (500μm). As displayed in **Figure 4**, aortic rings showed reduction in the number of sprouts upon safranal treatment as compared to the control. Day 8 showed an average of 227 micro vessels in rhVEGF induced untreated control whereas the safranal treated aortic ring sprouts only averaged 170 micro vessels despite rhVEGF induction.

### Angiogenic-Related Gene Expression Profiling Upon Safranal Treatment

**Figure 5:**
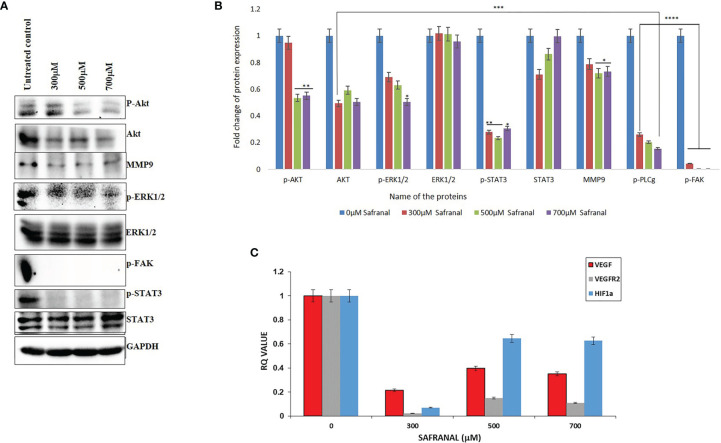
Safranal inhibited VEGF-induced angiogenesis signaling pathway in HCC cells. HepG2 cells were starved with 0.1% FBS overnight and then incubated with various concentrations of Safranal for 24 hrs, with VEGF (30 ng/ml). **(A)** The cell lysates were subjected to western blotting and probed with indicated antibodies, GAPDH was used as loading control. **(B)** Each band intensity was quantified to analyse the protein expression using ImageJ, normalized relative to their respective loading control bands. Values are expressed as ratio of untreated control in log fold. Statistical analysis was carried out in all experiments by student’s t-test using GraphPad Prism software and p < 0.05 was considered as statistically significant. *p < 0.05, **p < 0.01, ***p < 0.001, ****p < 0.0001. **(C)** Quantitative real-time PCR analysis shows that Safranal alters VEGF pathway family genes’, VEGF, VEGFR1, MMP3, HIF-1α, expressions in the HEPG2 cells.

Similar to the antagonistic effect on VEGF/VEGFR2 signalling, safranal affected the proangiogenic factors in HepG2 cells after 24 hrs of treatment in a dose dependent manner. The expression of p-AKT (Ser473), p-ERK1/2, p-FAK, and p-STAT3 were decreased by safranal under VEGF stimulus (**Figures 5A, B**). Further, matrix metalloproteinase-9 (MMP9) expressions were downregulated upon 24 hrs of safranal treatment, agreeing with the results of the invasive assays described earlier. Furthermore, we checked the effect of safranal on the transcription of VEGF and its functional cohorts, VEGFR2, and HIF-1α. Gene expression analysis using real time PCR showed a significant decrease of up to 80-90% of mRNA levels of VEGF, VEGFR2, and HIF-1α expression at a 300 μM dose of safranal treatment for 24 hours (**Figure 5C**). Using higher doses showed an irregular, but significant, decrease in the expression of these genes.

## Discussion

Safranal, the volatile component extracted from the stigma of the plant, *Crocus sativus* L; saffron, has been reported as potent anticancer and anti-inflammatory agent (27). This study attempts to provide an insight on safranal’s role in exerting antiangiogenic properties that could contribute to its anticancer potential. Safranal inhibited the growth of human endothelial cells, HUVEC, at the two tested doses with, and without, VEGF induction suggesting an antiproliferative capacity that may be mediated through VEGF and its receptors. VEGF and its receptors are key regulators of angiogenesis and vascular permeability that contribute heavily to the various stages of tumorigenesis (28). VEGF/VEGFR2 interaction acts as a key switch in the formation of new blood vessels that supply nutrients and oxygen to tumours (29). Here, safranal attenuated both VEGF secretion and VEGFR2 expression in HepG2 cells (**Figures 1B, C** **)**. VEGFR2 is the central receptor for VEGF-induced endothelial cell migration (30). We also successfully demonstrated the impact of safranal in cell migration *in vitro via* a wound healing assay. The wound area was completely closed by migrating untreated HUVEC cells (100%) whereas 50% and 72% of the wound area remained open using safranal doses of 500 μM and 700 μM respectively after 12 hours (**Figures 2A, B** **)**. As HUVEC endothelial cells are reported to migrate to extracellular matrix creating scaffolds aiding the formation of new blood vessels (31), this study, employs the Matrigel tube formation assay to mimic that sprouting of blood vessels in angiogenesis. In this assay safranal significantly (p<0.01) reduced the quantity (number of junctions) and quality (length of segments) (**Figures 2C–E**) of capillary-tube like structures formed after 8 hrs of safranal exposure and supported the anti-proliferative effect on HUVEC cells as discussed earlier. Tumor angiogenesis and metastasis requires many signalling circuits which involves invasion and the crosstalk between the environment of tumor and host cells (32). As shown in **Figure 3**, safranal inhibited the movement of HepG2 cells through the Boyden chamber without (crystal violet) and with (DAPI blue) extracellular matrix, thereby attesting to its anti-migratory and anti-invasive response in the presence of a chemoattractant. Sprouting of micro vessels *ex vivo* using the rat aortic ring angiogenesis assay can be used as a model for VEGF induced biological event (33). Morphological alterations were detected in aortic ring assays where treatment with safranal reduced the number of sprouting micro vessels in a time-dependent manner. These alterations were then quantitatively substantiated (**Figure 4**). Thanks to its inhibitory effects on angiogenesis, saffron was insinuated as a promising chemotherapeutic agent in breast cancer treatment (34). Collectively, such *ex vivo* results provide preliminary evidence of safranal’s possible chemotherapeutic, preventive, and adjunctive applications.

Tumor angiogenesis is associated with altered gene expression of angiogenic factors that are highly irregular compared to normal cells, hence forming vulnerable targets for cancer therapy (35). There was a remarkable reduction in various signalling molecules downstream of the VEGF autocrine pathway upon safranal treatment (**Figures 5A, B**). Typically, anti-angiogenesis treatments focus on central events like wound healing, migration, ECM interaction, infiltration, and invasion fuelling tumor growth (36). ERK and Akt activation by VEGF is a proven signalling pathway that enables cell migration, thereby facilitating vascular homeostasis and angiogenesis (37–39). Here, the expressions of p-AKT (Ser473) and p-ERK1/2 were decreased by safranal without affecting the expression of non-phosphorylated ERK1/2. Expression of AKT remained the same with a slightly higher expression in the untreated control (**Figures 5A, B**). This could be due to stimulation *via* rhVEGF as it can elevate the expression of many target molecules downstream to the autocrine pathway in HCC cells (40). Matrix metalloproteinases (MMPs) does proteolytic modulation of ECM and cell surfaces to facilitate the release of signal molecules like VEGF, thereby participating in metastasis and vasculature (41). Safranal reduced the phosphorylation of FAK at Tyr-397 and MMP9 protein (**Figures 5A, B**) which further strengthens its interplay between VEGF signalling. Numerous studies have shown that FAK inactivation impacts the expression of MMPs, augmenting invasion and tumor angiogenesis (42). Signal transducer and activator of transcription 3 (STAT3), is an important member of STATs family which has a major role in inflammation and human cancers. Irregular STAT3 signalling directly stimulates the expression of MMP9, promoting metastasis (43). Safranal inhibited the activation of STAT3 by blocking the phosphorylation at Tyr705 (**Figures 5A, B**). Many studies have proven that the inactivation of STAT3 attenuates key regulators participating in tumor angiogenic events like the migration of vascular cells and the sprouting of vessels, thereby enriching the tumor (44, 45).

Hypoxia takes center stage in tumor environments, leading to the stabilization of hypoxia-inducible factor, HIF-1αγ an important transcription factor that activates many hypoxia-response genes such as VEGF (46). Interestingly, there was a correlated decrease in the mRNA levels of HIF-1α and VEGF (**Figure 5C**), which clearly demonstrates the antiangiogenic effect of safranal. Safranal mediated inactivation of AKT/ERK axis could have manifested the reduction in the HIF-1α mRNA (47). The mRNA level of VEGFR2 is significantly higher in HCC as compared to non-tumor cells (48). From the qPCR data, safranal significantly reduces the mRNA expression of VEGFR2 (**Figure 5C**). This must be due to decreased VEGF synthesis which must have auto regulated the expression of its receptor, VEGFR2. The higher expression of VEGF and its receptors in HCC have been an encouraging signal towards a possible targeted therapy (49).

Taken together, the data presented here suggests that safranal has a pleiotropic effect where it targets multiple key regulators of tumor angiogenesis making them major candidates for potential anti-angiogenic therapy (50). Safranal significantly affects the strong interplay of HCC cell, endothelial cell, and multiple signalling molecules involved in tumor angiogenesis. Being the natural food ingredient of a spice, safranal may be a promising candidate for developing targeted, non-toxic, chemotherapeutic agents for cancer treatment.

## Data Availability Statement

The original contributions presented in the study are included in the article/supplementary material. Further inquiries can be directed to the corresponding author.

## Ethics Statement

The animal study was reviewed and approved by UAE University Animal Ethics Committee.

## Author Contributions

AAm designed the study and supervised all experiments. AAb and CM performed the experiments and did the statistical analysis. AAb and CM wrote the first draft of the manuscript. All authors contributed to the editing of the revised manuscript and approved the manuscript.

## Funding

This study was supported by UAEU College grant and ZCHS 31R174 for AAm.

## Conflict of Interest

The authors declare that the research was conducted in the absence of any commercial or financial relationships that could be construed as a potential conflict of interest.

## Publisher’s Note

All claims expressed in this article are solely those of the authors and do not necessarily represent those of their affiliated organizations, or those of the publisher, the editors and the reviewers. Any product that may be evaluated in this article, or claim that may be made by its manufacturer, is not guaranteed or endorsed by the publisher.
