# Supplementary material for: Safranal Inhibits Angiogenesis via Targeting HIF-1α/VEGF Machinery: In Vitro and Ex Vivo Insights
Source: Front Oncol. 2022 Feb 2;11:789172. doi: 10.3389/fonc.2021.789172 (PMC8862147; doi:10.3389/fonc.2021.789172)
Supplement: Supplementary file 1 [file Table_1.pdf]

GENE	Forward Primer (5' TO 3')		Reverse Primer (5' TO 3')
HIF1A	CACCACAGGACAGTACAGGAT		CGTGCTGAATAATACCACTCACA
VEGF	ATCTTCAAGCCATCCTGTGTGC		CAAGGCCCACAGGGATTTTC
VEGFR2	AGGCAGCTCACAGTCCTAGAGC		GTCTTTTCCTGGGCACCTTCTA
